# Performance evaluation of an amplicon‐based next‐generation sequencing panel for *BRCA1* and *BRCA2* variant detection

**DOI:** 10.1002/jcla.23524

**Published:** 2020-08-19

**Authors:** Kuenyoul Park, Min Kyu Kim, Taegeun Lee, Jinyoung Hong, Hyun‐Ki Kim, Sunyoung Ahn, Young‐Jae Lee, Jisun Kim, Shin‐Wha Lee, Jong Won Lee, Woochang Lee, Sail Chun, Byung Ho Son, Kyung Hae Jung, Yong‐Man Kim, Won‐Ki Min, Sei‐Hyun Ahn

**Affiliations:** ^1^ Department of Laboratory Medicine, Asan Medical Center University of Ulsan College of Medicine Seoul Korea; ^2^ Division of Gynecologic Oncology, Department of Obstetrics and Gynecology, Samsung Changwon Hospital Sungkyunkwan University School of Medicine Changwon‐Si Korea; ^3^ Department of Laboratory Medicine Kangwon National University School of Medicine Chuncheon Korea; ^4^ Department of Obstetrics and Gynecology, Asan Medical Center University of Ulsan College of Medicine Seoul Korea; ^5^ Department of Surgery, Asan Medical Center University of Ulsan College of Medicine Seoul Korea; ^6^ Department of Oncology, Asan Medical Center University of Ulsan College of Medicine Seoul Korea

**Keywords:** amplicon‐based panel, *BRCA1*, *BRCA2*, evaluation, next‐generation sequencing

## Abstract

**Background:**

As next‐generation sequencing (NGS) technology matures, various amplicon‐based NGS tests for *BRCA1/2* genotyping have been introduced. This study was designed to evaluate an NGS test using a newly released amplicon‐based panel, AmpliSeq for Illumina BRCA Panel (AmpliSeq panel), for detection of clinically significant *BRCA* variants, and to compare it to another amplicon‐based NGS test confirmed by Sanger sequencing.

**Methods:**

We reviewed *BRCA* test results done by NGS using the TruSeq Custom Amplicon kit from patients suspected of hereditary breast/ovarian cancer syndrome (HBOC) in 2018. Of those, 96 residual samples with 100 clinically significant variants were included in this study using predefined criteria: 100 variants were distributed throughout the *BRCA1* and *BRCA2* genes. All target variants were confirmed by Sanger sequencing. Duplicate NGS testing of these samples was performed using the AmpliSeq panel, and the concordance of results from the two amplicon‐based NGS tests was assessed.

**Results:**

Ninety‐nine of 100 variants were detected in duplicate *BRCA1/2* genotyping using the AmpliSeq panel (sensitivity, 99%; specificity, 100%). In the discordant case, one variant (*BRCA1* c.3627dupA) was found only in repeat 1, but not in repeat 2. Automated nomenclature of all variants, except for two indel variants, was in consensus with Human Genome Variation Society nomenclature.

**Conclusion:**

Our findings confirm that the analytic performance of the AmpliSeq panel is satisfactory, with high sensitivity and specificity.

## Graphical abstract

One hundred target variants were distributed throughout the *BRCA1* and *BRCA2* genes. AmpliSeq for Illumina BRCA Panel was satisfactory with 99% sensitivity and 100% specificity. Visual confirmation was needed for adequate reporting of indel variants.

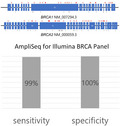

## 1 INTRODUCTION

In 1994, linkage analysis in large numbers of families identified *BRCA1* and *BRCA2* as genes associated with predisposition for hereditary breast/ovarian cancer syndrome (HBOC).^1^, ^2^ Approximately 5%‐10% and 20% of breast and ovarian cancer cases are considered hereditary tumors,^3^, ^4^ but only 25% of HBOC are associated with *BRCA1/2* pathogenic variants, which affect DNA repair mechanisms.^5^ Carriers with *BRCA1/2* pathogenic variants have a higher risk of developing breast cancer (60%‐85%) and ovarian cancer (15%‐40%) over their lifetime. ^6^, ^7^ In *BRCA*‐mutated patients, both intensive screening (including MRI) and prophylactic surgery or chemical treatment decrease cancer risk and overall mortality.^8^, ^9^, ^10^ Among triple‐negative breast cancer patients, platinum‐based chemotherapeutic agents are favorable for *BRCA1/2* variant carriers.^11^ Recently, poly ADP‐ribose polymerase (PARP) inhibitors were reported to improve prognosis in patients with *BRCA*‐mutated metastatic ovarian cancer.^12^ Collectively, these reports indicate that testing for *BRCA1/2* mutation plays a significant role in the choice of therapy, as well detection of the genetic cause.

Next‐generation sequencing (NGS) was introduced to clinical laboratories for multi‐gene and high‐throughput analysis.^13^ Subsequently, NGS has been developed as a powerful tool for detecting *BRCA1/2* variants.^14^, ^15^, ^16^, ^17^ Although the high performance and cost‐effectiveness of the NGS technique are well known, the diversity of NGS platforms, enrichment methods, and analytic pipelines represents a potential obstacle to implementation. Because amplicon‐based methods for enrichment have several strengths, including lower cost, shorter preparation time, and smaller quantities of input DNA in comparison with capture methods,^18^ several *BRCA1/2* NGS tests using amplicon methods have been developed.^19^, ^20^, ^21^, ^22^, ^23^ NGS‐based *BRCA1/2* variant tests have mainly been validated by Sanger sequencing, which is still considered to be the gold standard. This study was designed to evaluate the AmpliSeq for Illumina BRCA panel (AmpliSeq panel), an amplicon enrichment method for NGS testing, for detection of clinically significant *BRCA* variants, confirmed by Sanger sequencing, that were detected by another amplicon enrichment method, the TruSeq Custom Amplicon kit (TruSeq kit).

## 2 MATERIALS AND METHODS

The Institutional Review Board/Ethics Committee of Asan Medical Center waived the requirement for informed consent for this study (2019‐0044).

### 2.1 Sample selection and DNA extraction

**FIGURE 1:**
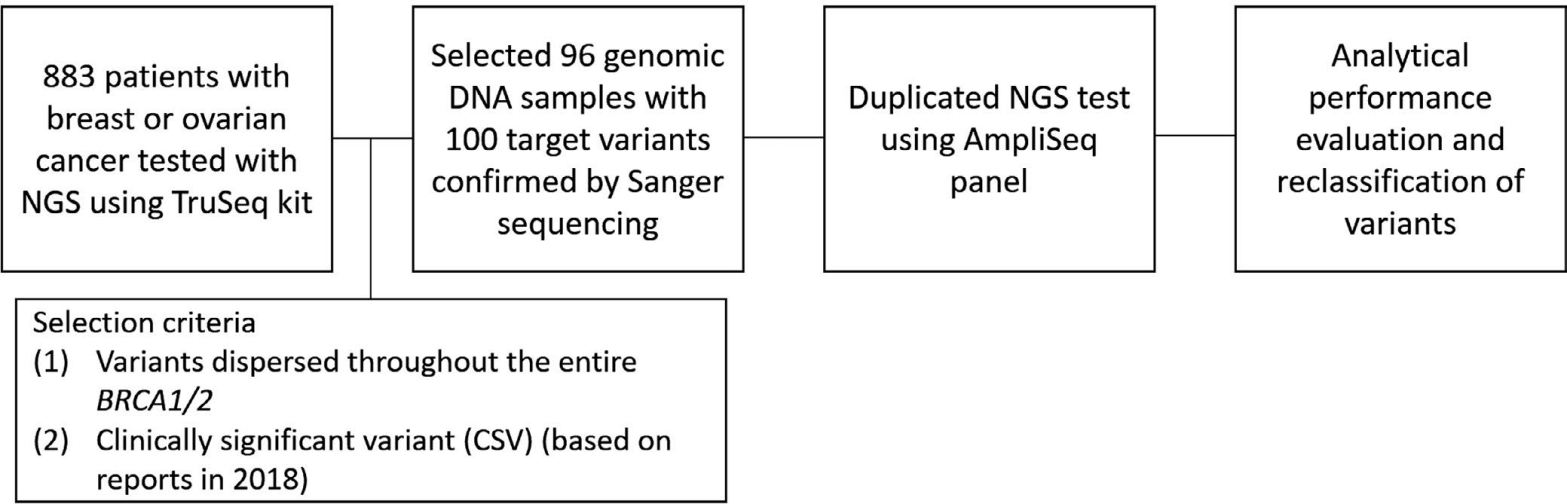
Schematic flowchart of the study design. Clinically significant variants included pathogenic variants, likely pathogenic variants, and variants of unknown significance. Abbreviation: TruSeq kit, TruSeq Custom Amplicon kit; AmpliSeq panel, AmpliSeq for Illumina BRCA panel

This study was performed at a single center. In 2018, 883 patients diagnosed with breast or ovarian cancer suspected to be HBOC were tested clinically for *BRCA1/2* variants by NGS using the TruSeq Custom Amplicon kit (Illumina) and Illumina MiSeqDx (Illumina) at our center. Genomic DNA was extracted from peripheral blood using the QIAGEN QIAamp DNA Mini Kit (QIAGEN). Of the reported variants from these 883 patients, 100 target variants were included in this study, based on the following criteria: (a) variants should be dispersed throughout the *BRCA1/2* genes, and (b) variants should be clinically significant (pathogenic, likely pathogenic, or variant of uncertain significance [VUS] based on the interpretation guideline from the American College of Medical Genetics and Genomics and the Association for Molecular Pathology).^24^ All target variants were confirmed by Sanger sequencing. Ninety‐six genomic DNA samples comprising 100 target variants were collected with anonymization. A schematic workflow of this study is shown in Figure 1.

### 2.2 AmpliSeq panel‐based NGS

A single NGS platform, MiSeqDx, was adopted for the detection of small indel and single‐nucleotide variants. The AmpliSeq for Illumina BRCA panel (Illumina), which contains 265 amplicons with average amplicon length of 98 bp, covers 22 404 base pairs, including all exons of *BRCA1/2*. Experiments using the AmpliSeq panel were performed in four separate batches containing 24 samples and repeated to confirm reproducibility.

### 2.3 Bioinformatic analysis

Human genome build 19 (hg19) was used for alignment. Analysis was performed with the Illumina MiSeq Reporter using the following software: DNA Amplicon BaseSpace Workflow 2.00, DNA Amplicon Workflow 3.23.7.3 + master, BWA‐MEM Whole‐Genome (aligner) 0.7.12‐r1039, Pisces Variant Caller 5.2.9.22, Illumina Annotation Engine 2.0.11‐0‐g7fb24a09, Bam Metrics v0.0.22, and SAMtools 1.2. Variants were filtered and annotated with Variant Studio. All variants were described according to the recommendation of the Human Genome Variation Society (https://www.hgvs.org/) using the reference transcript sequences of NM_007294.3 and NM_000059.3 for *BRCA1* and *BRCA2*, respectively. The target variants were confirmed using Integrative Genomic Viewer (IGV) (http://software.broadinstitute.org/software/igv). Along with the evaluation of this panel, the detected variants were reclassified based on the interpretation guidelines from the American College of Medical Genetics and Genomics and the Association for Molecular Pathology.^24^

### 2.4 Statistical analysis

To evaluate performance, the results from NGS using AmpliSeq panel were compared with the NGS results obtained using TruSeq kit and confirmed by Sanger sequencing. Sensitivity, specificity, negative predictive value, and positive predictive value were determined, and 95% confidence intervals were calculated using the efficient‐score method. The NGS test using AmpliSeq panel was performed in duplicate to examine reproducibility.

## 3 RESULTS

### 3.1 Target variants

**FIGURE 2:**
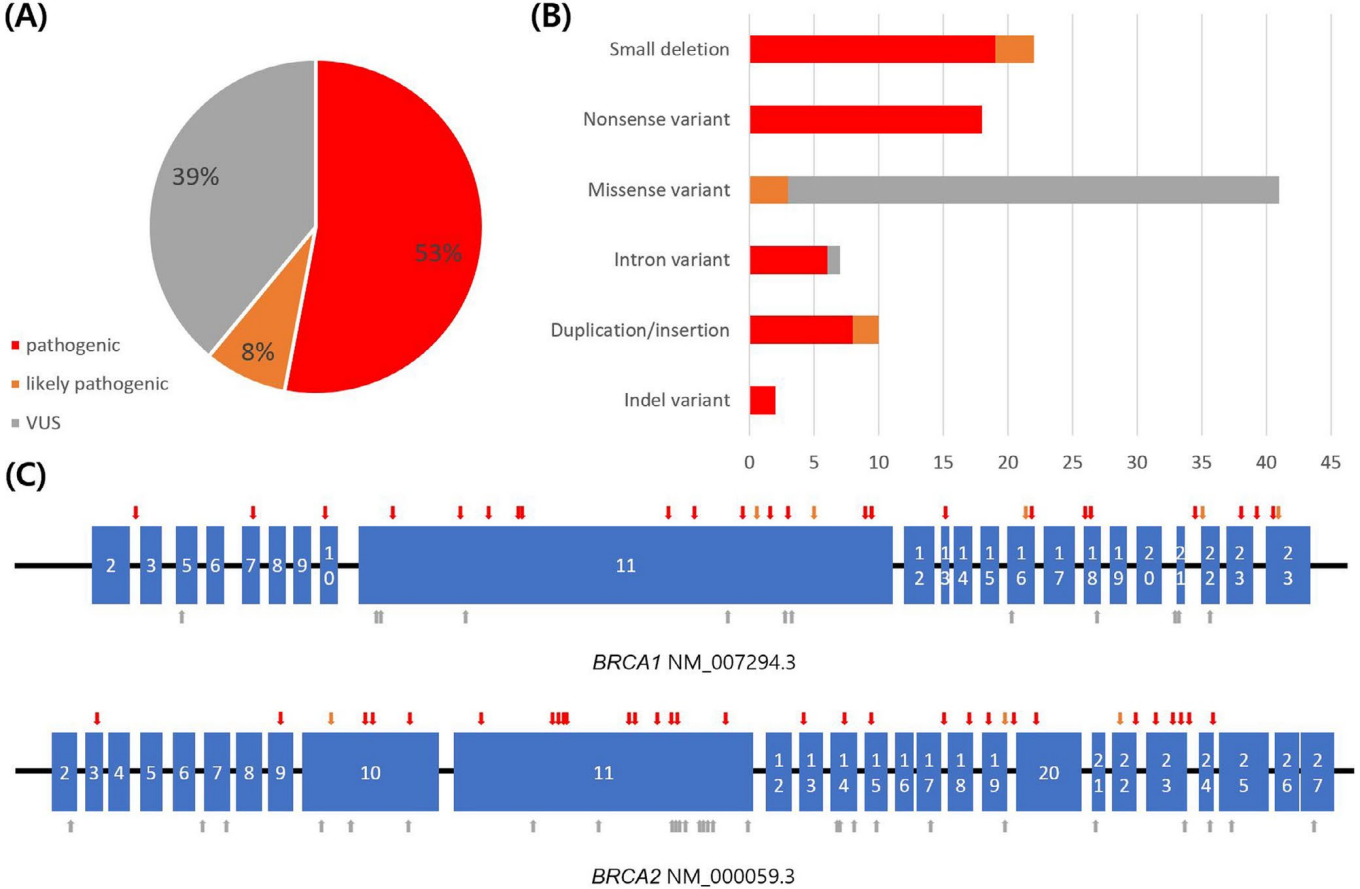
Types and distribution of clinically significant variants (n = 100) on *BRCA1* and *BRCA2* exons. A, Variant classifications, B, Variant types, C, Variant locations. Upper arrows indicate pathogenic or likely pathogenic variants, and lower arrows indicate VUS. Exon 4 was omitted because of a revision made after the initial description. Abbreviation: VUS, variants of unknown significance

We analyzed a total of 100 variants, comprising 66 single‐nucleotide variants and 34 indel variants. Characteristics of these variants are shown in Figure 2, and all variants are listed in Table S1. Only two variants (*BRCA1* c.5496_5506delinsA, *BRCA2* c.9309A > G) were found in two different samples, and all of the other variants were found in one sample.

### 3.2 Technical performance

A total of eight runs were performed: four batches, each containing 24 samples, were repeated. The quality control (QC) parameters of sequencing using the AmpliSeq panel were acceptable for all runs. The specific values of QC parameters are listed in Table 1.

**TABLE 1:** Run statistics of sequencing using AmpliSeq for Illumina BRCA

	Batch 1		Batch 2		Batch 3		Batch 4	
	Repeat 1	Repeat 2	Repeat 1	Repeat 2	Repeat 1	Repeat 2	Repeat 1	Repeat 2
On‐target reads, %	96.65	96.57	96.65	96.53	96.68	96.55	96.63	96.48
Percent Q30 bases	96.41	94.73	94.57	96.10	95.81	94.39	95.82	95.86
Coverage at 20X, %	100.00	100.00	100.00	100.00	100.00	100.00	100.00	100.00
Uniformity of base coverage at 0.2, %	99.98	100.00	99.95	100.00	100.00	100.00	100.00	100.00
Average depth per sample (min, max)	1410.5 (1085.9, 1838.6)	1432.2 (776.5, 3466.3)	1462.1 (1075.6, 1950.6)	1476.4 (928.6, 1915.8)	1481.0 (996.8, 2101.7)	1778.1 (1367.1, 3008.8)	1521.6 (963.6, 2386.0)	1569.1 (1059.4, 1931.6)

### 3.3 Analytical performance

All target variants except one were successfully detected by the AmpliSeq panel. Sensitivity, specificity, positive predictive value, and negative predictive value were 99%, 100%, 100%, and ~100% (95% confidence interval: 93.8%‐99.90%, 100.0%‐100.0%, 95.3%‐100%, and 100%‐100%), respectively, between the two NGS kits (Table 2). In *BRCA1*, one small duplication variant was not called from repeat 2 of one sample in batch 4. No discordant variants were found in *BRCA2*. Reproducibility of the AmpliSeq panel was 99.0% (100.0% for batches 1%‐3% and 95.8% for batch 4).

**TABLE 2:** Concordance of target variants between AmpliSeq for illumina BRCA and the TruSeq Custom Amplicon kit confirmed by Sanger sequencing

AmpliSeq panel	TruSeq Custom Amplicon kit confirmed by Sanger sequencing		Notes
	Detected	Not detected	
Detected	99 true positives	0 false positive	99% sensitivity (95% CI: 93.8%‐99.90%)
Not detected	1 false negative	22.4 Mbp true negatives	100% specificity (95% CI: 100%‐100%)

Note

In one false‐negative case, the variant was found in repeat 1 of AmpliSeq panel testing, but not called in repeat 2 due to low variant allele frequency.

**FIGURE 3:**
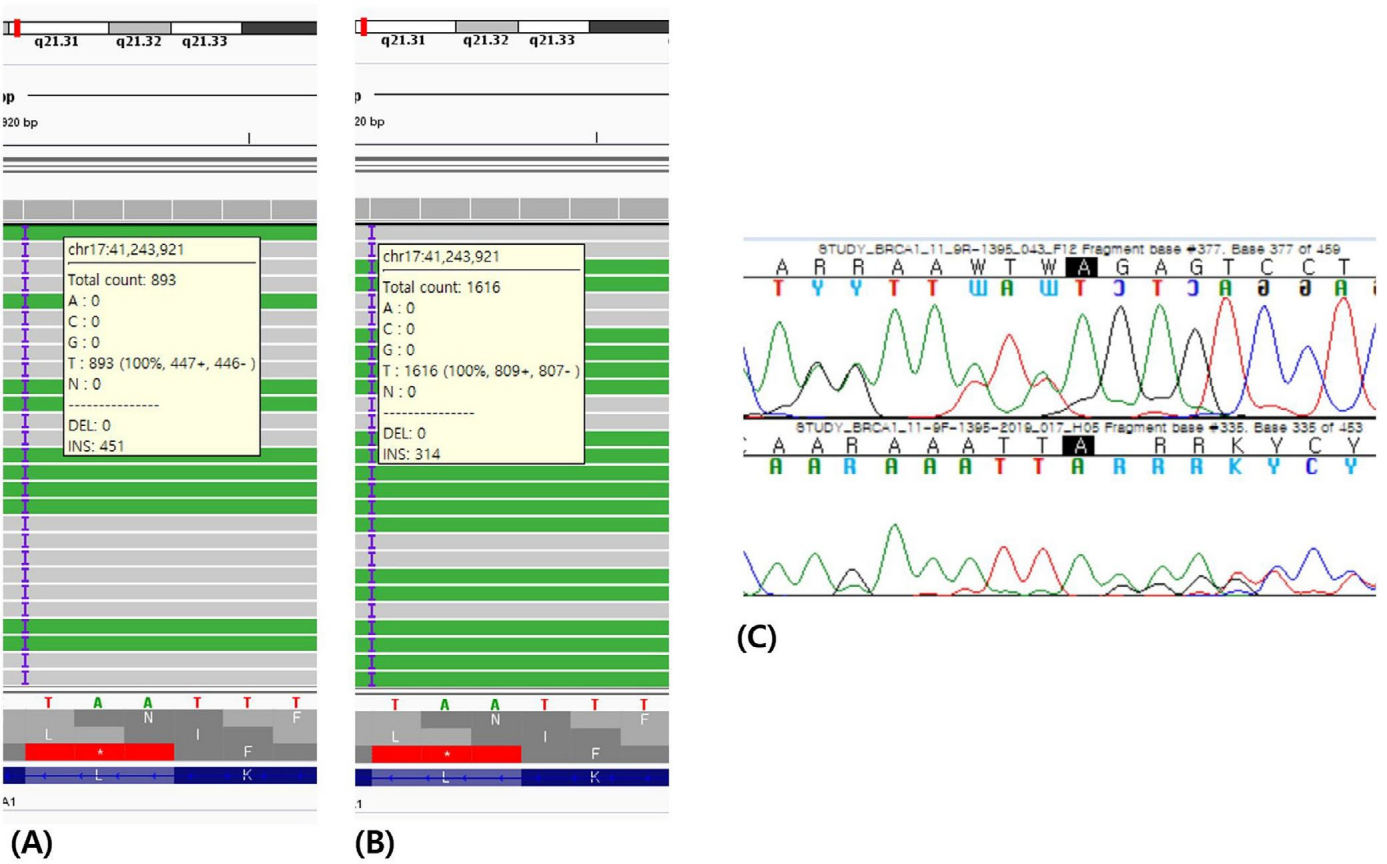
Integrative genomic viewer and chromatogram of a duplication variant from a discordant case. A, First repeat of AmpliSeq panel‐based NGS. B, Second repeat of AmpliSeq panel‐based NGS. C, Chromatogram of Sanger sequencing

In the discordant case, one variant (*BRCA1* c.3627dupA) was called only in repeat 1, but not in repeat 2. However, the variant was visible with low variant allele frequency (VAF) (19.4%) (Figure 3). After detailed review, we determined that all sixteen variants except one (from another sample in the same batch) were called: The exceptional case was detectable only on IGV due to low VAF. These observations suggested a possible error in sample preparation.

### 3.4 Variant annotation

After reclassification, eight variants from 39 target variants previously classified as VUS were designated as benign or likely benign. All were missense variants: five in *BRCA1* and four in *BRCA2*. Reclassification was mainly due to observations with a pathogenic variant. Thus, 31 variants remained as VUS. These are also listed in Table S1.

The nomenclature of all but two of the detected target variants was consistent with HGVS recommendations. In the two exceptional cases, indel variants (*BRCA1* c.922_924delAGCinsT and *BRCA1* c.5496_5506delinsA) were detected as two individual variants (one insertion variant and one deletion variant). These variants were observed in *cis* after sequence confirmation on IGV and were therefore reclassified as single indel variants.

## 4 DISCUSSION

The genetic diagnosis of breast and ovarian cancer is crucial for genetic counseling, surveillance, and tailored treatment. NGS‐based variant testing has emerged as a powerful tool for *BRCA1/2* gene testing. Therefore, several studies have validated NGS techniques for *BRCA1/2* testing. This study is the first to validate the performance of the AmpliSeq for Illumina BRCA panel for clinical application. Based on a comparison with the TruSeq kit confirmed by Sanger sequencing, our findings suggest that the analytical performance of the AmpliSeq panel is acceptable for detection of *BRCA1/2* variants.

In this study, the target variants were well dispersed throughout *BRCA1/2*. Germline variants of *BRCA1/2* are well known for their wide distribution.^25^, ^26^ Subsequently, widely distributed variations with visual confirmation are needed to adequately evaluate the performance of *BRCA1/2* variant testing. However, a functional study reported that more variants occur in the RING domain, exon 11‐13, and the BRAT domain of *BRCA1* ^27^; consistent with that, almost all *BRCA1* target variants (97.5%) were in these regions in this study.

The sensitivity and specificity of the AmpliSeq panel were 99% and 100%, respectively, and the one discordant case was probably due to a mistake in sample preparation. Therefore, this panel was nearly equivalent to the previously adopted NGS kit used for comparison, as well as Sanger sequencing. Moreover, the high reproducibility of the panel demonstrated its reliability. Other validation studies regarding NGS‐based *BRCA* testing revealed analytical specificity of 95.9%‐100% and analytical sensitivity of 100%.^19^, ^20^, ^28^ Collectively, these findings confirm the high performance of NGS‐based *BRCA1/2* testing.

In regard to the error in sample preparation, we note that the QC results from this run were acceptable. To avoid such errors, detected variants should be compared with variants found in other samples from the same run, and abnormal samples should be re‐examined. Other validation studies reported limitations due to technical factors such as low average coverage depth.^19^, ^28^ In addition to those sources of errors, procedural errors, such as in our case, do occur (albeit rarely) in the clinical laboratory. Therefore, this report emphasizes the need for clinical laboratories to make their best efforts to decrease errors in procedures.

In this study, indel variants were separated into insertion and deletion variants, mandating visual confirmation of whether the two variants were in *cis* or *trans*. Variant calling errors frequently arise for indel variants. Accordingly, we need to confirm all variants manually for accurate reporting of indel variants.

Our results indicated that the panel performed well but was limited by the low abundance of copy number variations (CNVs). In the Korean population, CNVs are less frequent than in other populations^29^, ^30^; the CNV frequency in Korean familial breast cancer patients ranges from 0.44% to 0.8%.^31^, ^32^ However, a novel NGS test for detection of CNVs in *BRCA1/2* is needed. Second, because this study was not a diagnostic cohort study, its clinical validity could not be investigated. However, we could adequately evaluate the analytic performance of this panel because we chose target variants widely dispersed throughout *BRCA1/2*.

In conclusion, this study shows that the analytic performance of AmpliSeq panel is satisfactory, with high sensitivity and specificity. Therefore, the AmpliSeq panel performs sufficiently well to be implemented in the clinical laboratory for detection of *BRCA1/2* variants. Further improvement in testing and bioinformatic platforms will be required to overcome the remaining limitations with regard to detection of CNVs detection and calling and annotation of indel variants.

## CONFLICT OF INTEREST

The authors thank Illumina, Inc for providing some library preparation kits and sequencing reagents.

## Supporting information

Tab S1
