# Supplementary material for: Performance evaluation of an amplicon‐based next‐generation sequencing panel for BRCA1 and BRCA2 variant detection
Source: J Clin Lab Anal. 2020 Aug 19;34(12):e23524. doi: 10.1002/jcla.23524 (PMC7755770; doi:10.1002/jcla.23524)
Supplement: Supplementary file 1 — Tab S1 [file JCLA-34-e23524-s001.docx]

**Table S1. Characteristics of target variants detected by next-generation sequencing using the TruSeq Custom Amplicon kit and confirmed by Sanger sequencing.**

| No. | gene | Nucleotide change | Amino acid change | Interpretation | Reinterpretation (if reclassified) | Variant type |
| --- | --- | --- | --- | --- | --- | --- |
| 1 | BRCA1 | c.81-9C>G | unknown | Pathogenic |  | Splicing |
| 2 | BRCA1 | c.154C>T | p.Leu52Phe | VUS | Benign/likely benign | Missense |
| 3 | BRCA1 | c.390C>A | p.Tyr130* | Pathogenic |  | Nonsense |
| 4 | BRCA1 | c.616C>T | p.Gln206* | Pathogenic |  | Nonsense |
| 5 | BRCA1 | c.811G>A | p.Val271Met | VUS | Benign/likely benign | Missense |
| 6 | BRCA1 | c.824G>A | p.Gly275Asp | VUS | Benign/likely benign | Missense |
| 7 | BRCA1 | c.922_924delAGCinsT | p.Ser308* | Pathogenic |  | Deletion-insertion |
| 8 | BRCA1 | c.1354delG | p.Val452* | Pathogenic |  | Small deletion |
| 9 | BRCA1 | c.1357G>C | p.Glu453Gln | VUS |  | Missense |
| 10 | BRCA1 | c.1511dupG | p.Lys505* | Pathogenic |  | Duplication |
| 11 | BRCA1 | c.1716delA | p.Glu572Aspfs*16 | Pathogenic |  | Deletion |
| 12 | BRCA1 | c.1729G>T | p.Glu577* | Pathogenic |  | Nonsense |
| 13 | BRCA1 | c.2678dupA | p.Lys894Glufs*9 | Pathogenic |  | Duplication/insertion |
| 14 | BRCA1 | c.2830delT | p.Cys944Valfs*56 | Pathogenic |  | Deletion |
| 15 | BRCA1 | c.3052A>G | p.Asn1018Asp | VUS |  | Missense |
| 16 | BRCA1 | c.3157delG | p.Glu1053Lysfs*9 | Pathogenic |  | Deletion |
| 17 | BRCA1 | c.3231delG | p.Pro1078Glnfs*3 | Likely pathogenic |  | Deletion |
| 18 | BRCA1 | c.3329delA | p.Lys1110Serfs*7 | Pathogenic |  | Deletion |
| 19 | BRCA1 | c.3403C>G | p.Gln1135Glu | VUS |  | Missense |
| 20 | BRCA1 | c.3442delG | p.Glu1148Argfs*7 | Pathogenic |  | Deletion |
| 21 | BRCA1 | c.3448C>T | p.Pro1150Ser | VUS | Benign/likely benign | Missense |
| 22 | BRCA1 | c.3627dupA | p.Glu1210Argfs*9 | Likely pathogenic |  | Duplication/insertion |
| 23 | BRCA1 | c.3954dupT | p.Gly1319Trpfs*11 | Pathogenic |  | Duplication/insertion |
| 24 | BRCA1 | c.3991C>T | p.Gln1331* | Pathogenic |  | Nonsense |
| 25 | BRCA1 | c.4335_4338dupAGAA | p.Gln1447Argfs*16 | Pathogenic |  | Duplication/insertion |
| 26 | BRCA1 | c.4729T>C | p.Ser1577Pro | VUS | Benign/likely benign | Missense |
| 27 | BRCA1 | c.4933delA | p.Arg1645Glyfs*13 | Likely pathogenic |  | Deletion |
| 28 | BRCA1 | c.4981G>T | p.Glu1661* | Pathogenic |  | Nonsense |
| 29 | BRCA1 | c.5080G>T | p.Glu1694* | Pathogenic |  | Nonsense |
| 30 | BRCA1 | c.5102_5103delTG | p.Leu1701Glnfs*14 | Pathogenic |  | Deletion |
| 31 | BRCA1 | c.5131A>G | p.Lys1711Glu | VUS |  | Missense |
| 32 | BRCA1 | c.5285G>C | p.Arg1762Thr | VUS |  | Missense |
| 33 | BRCA1 | c.5306A>G | p.Tyr1769Cys | VUS |  | Missense |
| 34 | BRCA1 | c.5333-2A>T | unknown | Pathogenic |  | Splicing |
| 35 | BRCA1 | c.5339T>C | p.Leu1780Pro | Likely pathogenic |  | Missense |
| 36 | BRCA1 | c.5372T>A | p.Val1791Glu | VUS |  | Missense |
| 37 | BRCA1 | c.5445G>A | p.Trp1815* | Pathogenic |  | Nonsense |
| 38 | BRCA1 | c.5467+1G>A | unknown | Pathogenic |  | Splicing |
| 39 | BRCA1 | c.5496_5506delinsA | p.Val1833Serfs*7 | Pathogenic |  | Deletion-insertion |
| 40 | BRCA1 | c.5509T>C | p.Trp1837Arg | Likely pathogenic |  | Missense |
| 41 | BRCA2 | c.52C>A | p.Asn18Ser | VUS |  | Missense |
| 42 | BRCA2 | c.276dupA | p.Ser93Ilefs*8 | Pathogenic |  | Duplication/insertion |
| 43 | BRCA2 | c.517-12C>A | unknown | VUS |  | Splicing |
| 44 | BRCA2 | c.623T>G | p.Val208Gly | VUS |  | Missense |
| 45 | BRCA2 | c.755_758delACAG | p.Asp252Valfs*24 | Pathogenic |  | Deletion |
| 46 | BRCA2 | c.964A>C | p.Lys322Gln | VUS | Benign/likely benign | Missense |
| 47 | BRCA2 | c.1056_1059delCTCA | p.Ser353Leufs*13 | Likely pathogenic |  | Deletion |
| 48 | BRCA2 | c.1196C>G | p.Thr399Ser | VUS |  | Missense |
| 49 | BRCA2 | c.1399A>T | p.Lys467* | Pathogenic |  | Nonsense |
| 50 | BRCA2 | c.1674_1680delTGATAAT | p.Ile558Metfs*13 | Pathogenic |  | Deletion |
| 51 | BRCA2 | c.1744A>C | p.Thr582Pro | VUS | Benign/likely benign | Missense |
| 52 | BRCA2 | c.1796_1800delCTTAT | p.Ser599* | Pathogenic |  | Small deletion |
| 53 | BRCA2 | c.2435delA | p.Asn812Ilefs*13 | Pathogenic |  | Deletion |
| 54 | BRCA2 | c.3220A>T | p.Ser1074Cys | VUS |  | Missense |
| 55 | BRCA2 | c.3599_3600delGT | p.Cys1200* | Pathogenic |  | Small deletion |
| 56 | BRCA2 | c.3623T>G | p.Leu1208* | Pathogenic |  | Nonsense |
| 57 | BRCA2 | c.3744_3747delTGAG | p.Ser1248Argfs*10 | Pathogenic |  | Deletion |
| 58 | BRCA2 | c.3847_3848delGT | p.Val1283Lysfs*2 | Pathogenic |  | Deletion |
| 59 | BRCA2 | c.4320A>C | p.Lys1440Asn | VUS |  | Missense |
| 60 | BRCA2 | c.4829_4830delTG | p.Val1610Glyfs*4 | Pathogenic |  | Deletion |
| 61 | BRCA2 | c.4936_4939delGAAA | p.Glu1646Glnfs*23 | Pathogenic |  | Deletion |
| 62 | BRCA2 | c.5351dupA | p.Asn1784Lysfs*3 | Pathogenic |  | Duplication/insertion |
| 63 | BRCA2 | c.5539G>A | p.Ala1847Thr | VUS |  | Missense |
| 64 | BRCA2 | c.5554G>A | p.Val1852Ile | VUS |  | Missense |
| 65 | BRCA2 | c.5576_5579delTTAA | p.Ile1859Lysfs*3 | Pathogenic |  | Deletion |
| 66 | BRCA2 | c.5590G>A | p.Asp1864Asn | VUS |  | Missense |
| 67 | BRCA2 | c.5699C>G | p.Ser1900* | Pathogenic |  | Nonsense |
| 68 | BRCA2 | c.5784A>C | p.Glu1928Asp | VUS |  | Missense |
| 69 | BRCA2 | c.5969A>C | p.Asp1990Ala | VUS | Benign/likely benign | Missense |
| 70 | BRCA2 | c.6029T>G | p.Val2010Gly | VUS |  | Missense |
| 71 | BRCA2 | c.6122C>A | p.Ser2041Tyr | VUS |  | Missense |
| 72 | BRCA2 | c.6447_6448dupTA | p.Lys2150Ilefs*19 | Pathogenic |  | Duplication/insertion |
| 73 | BRCA2 | c.6745G>A | p.Ala2249Thr | VUS |  | Missense |
| 74 | BRCA2 | c.6952C>T | p.Arg2318* | Pathogenic |  | Nonsense |
| 75 | BRCA2 | c.7051G>A | p.Ala2351Thr | VUS |  | Missense |
| 76 | BRCA2 | c.7052C>G | p.Ala2351Gly | VUS | Benign/likely benign | Missense |
| 77 | BRCA2 | c.7258G>T | p.Glu2420* | Pathogenic |  | Nonsense |
| 78 | BRCA2 | c.7411A>G | p.Thr2471Ala | VUS |  | Missense |
| 79 | BRCA2 | c.7480C>T | p.Arg2494* | Pathogenic |  | Nonsense |
| 80 | BRCA2 | c.7522G>A | p.Gly2508Ser | VUS |  | Missense |
| 81 | BRCA2 | c.7901T>A | p.Met2634Lys | VUS |  | Missense |
| 82 | BRCA2 | c.7976+1G>A | unknown | Pathogenic |  | Splicing |
| 83 | BRCA2 | c.8298_8299dupAC | p.Pro2767Hisfs*11 | Pathogenic |  | Duplication/insertion |
| 84 | BRCA2 | c.8363G>A | p.Trp2788* | Pathogenic |  | Nonsense |
| 85 | BRCA2 | c.8486A>G | p.Gln2829Arg | Likely pathogenic |  | Missense |
| 86 | BRCA2 | c.8487G>T | p.Gln2829His | VUS |  | Missense |
| 87 | BRCA2 | c.8488-1G>A | unknown | Pathogenic |  | Splicing |
| 88 | BRCA2 | c.8537_8538delAG | p.Glu2846Glyfs*22 | Pathogenic |  | Deletion |
| 89 | BRCA2 | c.8651A>G | p.Tyr2884Cys | VUS |  | Missense |
| 90 | BRCA2 | c.8803dupA | p.Met2935Asnfs*4 | Likely pathogenic |  | Duplication/insertion |
| 91 | BRCA2 | c.8951C>G | p.Ser2984* | Pathogenic |  | Nonsense |
| 92 | BRCA2 | c.8991T>G | p.Tyr2997* | Pathogenic |  | Nonsense |
| 93 | BRCA2 | c.9076C>T | p.Gln3026* | Pathogenic |  | Nonsense |
| 94 | BRCA2 | c.9105T>G | p.Tyr3035* | Pathogenic |  | Nonsense |
| 95 | BRCA2 | c.9110_9112delAAC | p.Gln3037del | VUS |  | Missense |
| 96 | BRCA2 | c.9117+1G>A | unknown | Pathogenic |  | Splicing |
| 97 | BRCA2 | c.9241G>A | p.Val3081Ile | VUS |  | Missense |
| 98 | BRCA2 | c.9253delA | p.Thr3085Glnfs*19 | Pathogenic |  | Deletion |
| 99 | BRCA2 | c.9309A>G | p.Ile3103Met | VUS |  | Missense |
| 100 | BRCA2 | c.10131A>C | p.Glu3377Asp | VUS |  | Missense |
